# Longitudinal Evidence for Attenuated Local-Global Deviance Detection as a Precursor of Working Memory Decline

**DOI:** 10.1523/ENEURO.0156-23.2023

**Published:** 2023-08-10

**Authors:** Yi-Fang Hsu, Chia-An Tu, Tristan A. Bekinschtein, Jarmo A. Hämäläinen

**Affiliations:** 1 Department of Educational Psychology and Counselling, National Taiwan Normal University, Taipei 106308, Taiwan; 2 Institute for Research Excellence in Learning Sciences, National Taiwan Normal University, Taipei 106308, Taiwan; 3 Department of Psychology, University of Cambridge, Cambridge CB2 3EB, United Kingdom; 4 Jyväskylä Centre for Interdisciplinary Brain Research, Department of Psychology, University of Jyväskylä, Jyväskylä 40014, Finland

**Keywords:** aging, auditory perception, electroencephalography (EEG), prediction errors, predictive coding

## Abstract

From the perspective of predictive coding, normal aging is accompanied by decreased weighting of sensory inputs and increased reliance on predictions, resulting in the attenuation of prediction errors in older age. Recent electroencephalography (EEG) research further revealed that the age-related shift from sensorium to predictions is hierarchy-selective, as older brains show little reduction in lower-level but significant suppression in higher-level prediction errors. Moreover, the disrupted propagation of prediction errors from the lower-level to the higher-level seems to be linked to deficient maintenance of information in working memory. However, it is unclear whether the hierarchical predictive processing continues to decline with advancing age as working memory. Here, we longitudinally followed a sample of 78 participants from three age groups (including seniors, adults, and adolescents) over three years’ time. Seniors exhibited largely preserved local processing [consisting of comparable mismatch negativity (MMN), delayed P3a, and comparable reorienting negativity (RON)] but significantly compromised global processing (consisting of suppressed frontocentral negativity and suppressed P3b) in the auditory local-global paradigm. These electrophysiological responses did not change with the passing of time, unlike working memory which deteriorated with advancing age. Correlation analysis further showed that these electrophysiological responses signaling prediction errors are indicative of concurrent working memory. Moreover, there was a correlation between earlier predictive processing and later working memory but not between earlier working memory and later predictive processing. The temporal asymmetry suggested that the hierarchy-selective attenuation of prediction errors is likely a precursor of working memory decline.

## Significance Statement

While predictive coding is postulated as a fundamental principle of brain function, little is known about its developmental trajectory in normal aging. This is in stark contrast with the large body of research on age-related decline in cognition such as working memory, which could be conceptualized as a derivative of the predictive coding mechanism. Here, we provided longitudinal evidence that the hierarchy-selective attenuation of prediction errors manifests as a stable feature while working memory deteriorates with advancing age. Correlation analysis further suggested that the attenuation of prediction errors is likely a precursor of working memory decline, rendering it a potential predictor of working memory deterioration before any clinically relevant symptoms would manifest in the aging population.

## Introduction

From the perspective of predictive coding, our perceptual system uses a hierarchical generative model to proactively represent the statistical structure of the environment (Friston, 2005, 2009; Egner et al., 2010; Feldman and Friston, 2010; for review, see Clark, 2013; Heilbron and Chait, 2018). This involves the matching of sensory inputs against a cascade of top-down predictions. The mismatch between the two creates prediction errors, which would be propagated forward to update the model of the environment.

Normal aging is reported to degrade precision of peripheral and central processing, leading to decreased weighting of sensory inputs and increased reliance on predictions (Moran et al., 2014; Wolpe et al., 2016; Chan et al., 2021). This would result in the attenuation of prediction errors in older age, manifested as reduced neural responses. Recent electroencephalography (EEG) research further showed that the age-related shift from sensorium to predictions is hierarchy-selective in the auditory local-global paradigm (Hsu et al., 2021). In the auditory local-global paradigm which orthogonally manipulated local (i.e., lower-level) and global (i.e., higher-level) regularities, a common finding is that local violations would elicit the mismatch negativity (MMN), P3a, and reorienting negativity (RON) signaling automatic processing of prediction errors and that global violations would elicit the frontocentral negativity and P3b signaling conscious processing of prediction errors (Bekinschtein et al., 2009; see also Wacongne et al., 2011; Chennu et al., 2013; El Karoui et al., 2015). Hsu et al. (2021) demonstrated that older brains show little reduction in the MMN but significant suppression in the P3b. Given the hierarchical structure of predictive processing, it seemed that the intact lower-level prediction errors originating from a restricted network at temporofrontal cortices (Rinne et al., 2000; Chennu et al., 2016; for review, see Alho, 1995) cannot properly accumulate to inform the computation of higher-level prediction errors at a brain-scale network (Tarkka et al., 1995; Polich, 2004, 2007; Patel and Azzam, 2005). Moreover, correlation analysis revealed that the disrupted propagation of prediction errors from the lower-level to the higher-level is linked to deficient maintenance of information in working memory.

However, it is unclear whether the hierarchy-selective attenuation of prediction errors advances with the passing of time, while it is well established that increased age is associated with reduction in the effectiveness of working memory, showing a decrease of 0.20–0.40 units per decade across adulthood (for review, see Salthouse, 1990, 1994). Studying the developmental trajectory of the hierarchical predictive processing alongside working memory would be the first step in uncovering the relationship between predictive coding (as a fundamental principle of brain function) and working memory (which could be conceptualized as a process of evidence accumulation (Parr and Friston, 2017) and evidence optimization (Trapp et al., 2021) from the perspective of Bayesian inference). Here, we longitudinally followed a sample of 78 participants from three age groups (including seniors, adults, and adolescents) over three years’ time to investigate how the hierarchical predictive processing might change alongside working memory in normal aging. Specifically, we examined whether there are any diminutions in the hierarchical predictive processing as well as working memory and whether their correlation persists with advancing age.

## Materials and Methods

### Participants

Participants were recruited from a cohort of 108 healthy volunteers in Hsu et al. (2021), including 36 seniors (age range = 55–82, mean = 65.31, SD = 7.03), 36 adults (age range = 19–27 years, mean = 21.17, SD = 2.01), and 36 adolescents (age range = 15–18, mean = 16.97, SD = 0.81), all reporting no history of neurologic, neuropsychiatric, visual, or hearing impairments. A total of 78 participants returned, including 27 seniors (age range = 58–85, mean = 68.44, SD = 7.18), 22 adults (age range = 22–29, mean = 24.26, SD = 1.65), and 29 adolescents (age range = 18–21, mean = 19.45, SD = 0.66). The follow-up interval between measurement points of time 1 and time 2 (i.e., T1 and T2) was around three years (day range = 977–1121, mean = 1053, SD = 36.26). Participants gave written informed consent and were paid for participation. The study was conducted in accordance with the Declaration of Helsinki and approved by the Research Ethics Committee at National Taiwan Normal University.

Participants provided us with information about their gender, handedness, level of education, and marital status (Table 1) before undergoing a neuropsychological evaluation of cognitive functions. Their working memory was measured with three subtests in Wechsler Adult Intelligence Scale-Fourth Edition (WAIS-IV), including Digit Span (comprising Forward, Backward, and Sequencing), Letter-Number Sequencing, and Arithmetic. Raw scores of the three subtests were converted to *z* scores and summed. Their depressive symptoms were measured with the Center for Epidemiological Studies-Depression (CES-D) scale (Radloff, 1977; Chien and Cheng, 1985), a 20-item self-report scale that asks one to rate how often over the past week he/she experienced symptoms associated with depression. Response options range from 1 [rarely or none of the time (<1 d)] to 4 [most or all of the time (5–7 d)] for each item. Their level of perceived stress was measured with the Perceived Stress Scale (PSS; Cohen et al., 1983; Chu, 2005), a 14-item self-report scale that asks about one’s feelings and thoughts during the last month. Response options range from 0 (never) to 4 (very often) for each item. All tests were administered and scored by trained personnel according to standard procedures.

**Table 1:** Demographic characteristics of the participants

		Seniors	Adults	Adolescents
Gender	Male	9	5	16
	Female	18	17	13
Handedness	Right	25	21	27
	Left	1	1	2
	Ambidextrous	1	0	0
Level of education	Primary	1	0	0
	Secondary	16	3	28
	Tertiary	10	19	1
Marital status	Single	3	21	29
	Married	19	1	0
	Divorced	1	0	0
	Widowed	4	0	0

### Stimuli

A total of 14 sinusoidal tones were generated using Sound Forge Pro 10.0 (Sony Creative Software Inc.). The duration of each tone was 50 ms (including 5-ms rise/fall times). The frequency of each tone was within the range of 261.626–987.767 Hz, matching the absolute frequency of a series of 14 natural keys on a modern piano (i.e., C4 D4 E4 F4 G4 A4 B4 C5 D5 E5 F5 G5 A5 B5; Table 2).

**Table 2:** Frequency (Hz) of each tone

	C4	D4	E4	F4	G4	A4	B4
Frequency	261.63	293.67	329.63	349.23	392.00	440.00	493.88
							
	C5	D5	E5	F5	G5	A5	B5
Frequency	523.25	587.33	659.26	698.46	783.99	880.00	987.77

**Figure 1.**
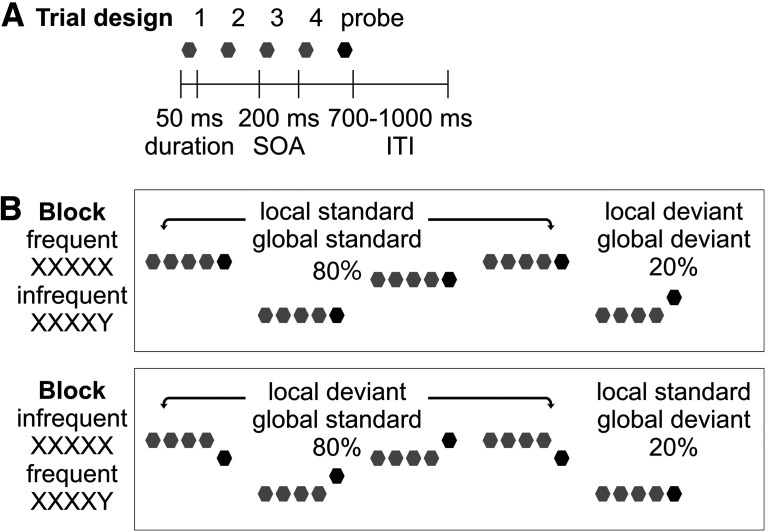
Schematic illustration of the auditory local-global paradigm. ***A***, Each trial contained five sinusoidal tones of 50 ms in duration presented with a 200-ms SOA, followed by a jittered ITI of 700–1000 ms. ***B***, Each block contained two different types of tone quintets, the first using the five-time repetition of the same tone (i.e., XXXXX) and the second with a change in the last tone (i.e., XXXXY). Half of the blocks contained frequent repetition with infrequent change in the last tone (upper: 80% XXXXX and 20% XXXXY) and half contained infrequent repetition with frequent change in the last tone (lower: 20% XXXXX and 80% XXXXY). The tone quintets are plotted on different lines to represent that we used 14 tones as stimuli to introduce variation in pitch. The last tone in each tone quintet (i.e., probe) is marked in black as the tone of interest. Participants were instructed to (1) identify infrequent trials and (2) count the number of infrequent trials, both of which were reported at the end of each block.

While previous research adopting the auditory local-global paradigm used two tones as stimuli (Bekinschtein et al., 2009; Wacongne et al., 2011; Chennu et al., 2013; El Karoui et al., 2015), here we used 14 tones as stimuli to introduce variation in pitch, which should decrease stimulus-specific effects (hence ensuring that neural responses indeed reflect prediction errors per se) and increase task difficulty (hence maximizing individual differences in task performance). Specifically, from the pool of 14 tones, two different types of tone quintets were created, the first using the five-time repetition of the same tone (i.e., the XXXXX type, which could be C4-C4-C4-C4-C4, B4-B4-B4-B4-B4, etc.) and the second with a change in the last tone (i.e., the XXXXY type, which could be A5-A5-A5-A5-C4, D5-D5-D5-D5-B4, etc.). For blocks of frequent repetition and infrequent change (Fig. 1*B*, upper), 80% of the trials were randomly sampled from the XXXXX type and 20% from the XXXXY type. For blocks of infrequent repetition and frequent change (Fig. 1*B*, lower), 20% of the trials were randomly sampled from the XXXXX type and 80% from the XXXXY type. The last tone in each tone quintet (i.e., probe) was the tone of interest, which can be either a local standard and a global standard (i.e., XXXXX in blocks of frequent XXXXX), a local deviant and a global deviant (i.e., XXXXY in blocks of frequent XXXXX), a local standard and a global deviant (i.e., XXXXX in blocks of frequent XXXXY), or a local deviant and a global standard (i.e., XXXXY in blocks of frequent XXXXY).

### Procedures

A total of 1000 tone quintets were presented in eight blocks in randomized order. Half of the blocks contained frequent repetition and infrequent change in the last tone (i.e., Fig. 1*B*, upper: 80% XXXXX and 20% XXXXY) and half contained infrequent repetition and frequent change in the last tone (i.e., Fig. 1*B*, lower: 20% XXXXX and 80% XXXXY). Both block types presented a local regularity where the last tone could be identical to or different from the four preceding tones (hence the local standard and deviant) and a global regularity where one tone quintet could be more or less common than the other (hence the global standard and deviant). In each block, the number of tone quintets varied between 110 and 140, where the number of frequent tone quintets varied between 88 and 112 and the number of infrequent tone quintets varied between 22 and 28 to maintain the 80:20 frequency ratio. Each block started with at least 20 frequent tone quintets to establish the global regularity before the first infrequent tone quintet appeared.

A gray fixation cross against black background remained on the screen for the duration of each block, viewed from a distance of 120 cm. Each trial contained five sinusoidal tones presented with a 200-ms stimulus onset asynchrony (SOA), followed by a jittered intertrial interval (ITI) of 700–1000 ms (Fig. 1*A*). Participants were instructed that each block contained two different types of tone quintets (i.e., XXXXX and XXXXY). They should (1) identify infrequent trials and (2) count the number of infrequent trials, both of which were reported at the end of each block. This task was implemented to ensure that we can observe not only the local prediction errors which are automatic in nature but also the global prediction errors which require conscious awareness (Bekinschtein et al., 2009). The whole experiment took around 29 min (i.e., 1000 trials × 1700 ms). E-prime version 2.0 (Psychology Software Tools) was used for stimulus presentation. Stimulation was randomized individually for each participant and delivered binaurally via headphones (Sennheiser PX200-II) with an intensity of maximum 82 dB (56–82 dBA; 65–82 dBC).

### EEG recording and preprocessing

EEG was recorded from 62 sintered Ag/AgCl electrodes on a Neuroscan quik-cap according to the extended 10–20 system. The ground electrode was placed at AFz and the reference electrode was placed between Cz and CPz. Eye movements were monitored by additional four electrodes placed above and below the left eye and at the outer canthi of both eyes, which were bipolarized online to yield vertical and horizontal electro-oculogram (EOG), respectively. All signals were amplified and online filtered at 0.1–100 Hz with the Neuroscan Synamps 2 amplifier (Compumedics Neuroscan) and sampled at 500 Hz.

Epochs extended from −900 to 498 ms relative to probe onset, using a −100 to 0 ms preprobe baseline. Ocular artifact correction was conducted with independent component analysis (ICA) in EEGlab (Delorme and Makeig, 2004) using the runica algorithm. ICs capturing blinks and horizontal eye movements were determined by visual inspection, pruning out one to three components (in T1) and zero to three components (in T2) for each participant. The data were recomputed to average reference, highpass-filtered at 0.5 Hz, and lowpass-filtered at 20 Hz. Epochs containing voltage deviations exceeding ±100 μV relative to baseline at any of the electrodes were rejected.

### Statistical analysis of behavioral measures

We performed a 3 (age group: seniors, adults, adolescents) × 2 (measurement point: T1, T2) repeated measures ANOVA on cognitive functions (including working memory, depressive symptoms, and level of perceived stress) as well as task performance (including identification accuracy and counting errors). Games–Howell pairwise comparisons were used in *post hoc* analysis for unequal sample sizes.

### Statistical analysis of the event-related potentials (ERPs)

Local effect was defined as the ERP difference between all local deviants and local standards (i.e., deviant − standard, averaged across global manipulation). Global effect was defined as the ERP difference between all global deviants and global standards (i.e., deviant − standard, averaged across local manipulation).

Since our previous research using cluster-based permutation statistics (Hsu et al., 2021) already showed that local effect manifested as the MMN, P3a, and RON and that global effect manifested as the frontocentral negativity and P3b, here we used a temporal principal component analysis (PCA) to quantify these components of interest, which has been considered an effective linear reduction method for multivariate ERP data since it was first introduced (Ruchkin et al., 1964; Donchin, 1966; Möcks, 1988a, b; Duffy et al., 1992; Chapman and McCrary, 1995; Dien, 1998; Picton et al., 2000; Dien and Frishkoff, 2005; for review, see Kayser and Tenke, 2003; Dien, 2012). It statistically decomposes the ERP waveforms into constituent building blocks, which affords data-driven ERP component measures compared with other conventional methods (Kayser et al., 1998; Beauducel et al., 2000; Kayser and Tenke, 2006). Moreover, it is not as susceptible to the influences of high-frequency noises and low-frequency drifts in the data as other conventional methods (Luck, 2005). The PCA was conducted in SPSS 23, separately for the difference waveforms of local and global effects. The data used for component extraction included data from all electrodes of each participant. Covariance matrix and Promax rotation were used. All components accounting for a total of 99% of the variance (maximum iterations for convergence = 500) were included in the rotation (Promax κ = 4). The decomposition provided a set of time-variant component loadings reflecting the contribution of each temporal component to the voltage at each time point and a set of time-invariant component scores (calculated using Bartlett method) representing the contribution of each temporal component to the ERP waveforms which can be subject to inferential statistics (Van Boxtel, 1998).

Components of interest were identified on the basis of the component loading latencies and the component score topographies. Specifically, we looked for PCs whose component loading latencies and component score topographies match the latencies and topographies of the MMN/P3a/RON and the frontocentral negativity and P3b in our previous research (Hsu et al., 2021). Concerning local effects, we identified two PCs corresponding to the MMN (i.e., PC13 and PC9), two PCs corresponding to the P3a (i.e., PC2 and PC11), and one PC corresponding to the RON (i.e., PC1). Concerning global effects, we identified two PCs corresponding to the frontocentral negativity (i.e., PC16 and PC4) and one PC corresponding to the P3b (i.e., PC1). Two PCs can be identified as the same component because they showed similar latencies and topographies, but loaded differently for the conditions.

The component scores were averaged across three electrodes showing the most negative/positive responses independent of experimental manipulation to serve as objective representatives of the components (Table 3). The advantage of averaging three maximum electrodes was twofold. First, it increased the signal-to-noise ratio of the components. Second, it avoided the problems inherited in the analysis of predefined areas that took an average of multiple electrodes over predefined regions, which might not correspond to the true topographies in the experiment. We performed a 3 (age group: seniors, adults, adolescents) × 2 (measurement point: T1, T2) repeated measures ANOVA on the aforementioned component scores with Bonferroni correction, using an α level of 0.006 (i.e., 0.05/8). Games–Howell pairwise comparisons were used in *post hoc* analysis for unequal sample sizes.

**Table 3:** Percentage of variance accounted for, peak latency, and three maximum electrodes of each PC

	Local effects					Global effects		
	MMN	MMN	P3a	P3a	RON	FN	FN	P3b
	PC13	PC9	PC2	PC11	PC1	PC16	PC4	PC1
% of variance accounted for	1.45%	1.93%	18.63%	1.64%	25.85%	0.83%	2.79%	50.57%
Peak latency (ms)	108	156	224	308	456	150	190	456
Three maximum electrodes	F4 F2 FC4	Fz F2 F1	FCz Cz FC2	FCz Cz FC2	Fz F1 FC1	F4 FC6 FC3	F4 F2 Fz	Pz P1 POz

### Correlation analysis

To explore the relation between the ERPs and cognitive functions, we conducted partial correlations between the local and global effects (including the aforementioned component scores of the MMN/P3a/RON as well as the frontocentral negativity and P3b) versus working memory, where participants’ scores on CES-D and PSS in both measurement points were controlled for to remove the potential influences of psychological factors (i.e., depressive symptoms and level of perceived stress) on working memory.

## Results

### Cognitive functions

**Figure 2.**
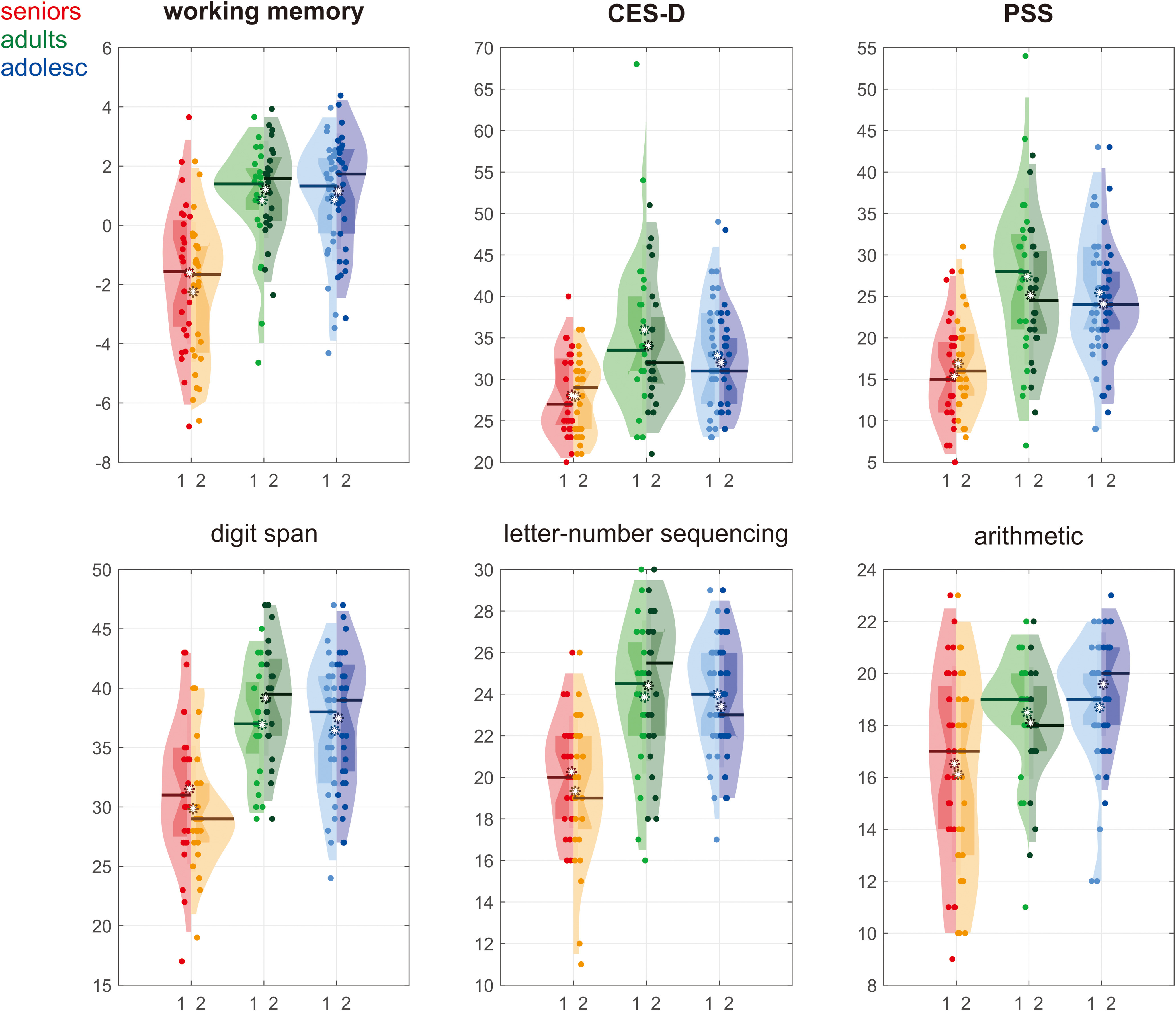
Cognitive functions measured with working memory subtests, CES-D, and PSS in each age group (i.e., seniors, adults, adolescents) and measurement point (i.e., T1, T2), visualized using violin plots (Legouhy, 2022). The white asterisk indicates the mean, the central mark indicates the median, and the edges of the box indicate the 25th and 75th percentiles, respectively.

A significant age group × measurement point interaction was found on working memory (*F*_(2,75)_ = 5.46, *p* = 0.006, η_p_^2^ = 0.13) but not depressive symptoms or level of perceived stress (Fig. 2, visualized using violin plots (Legouhy, 2022)).

For working memory, simple effects of age groups were found in both T1 (*F*_(2,77)_ = 11.32, *p* < 0.001) and T2 (*F*_(2,77)_ = 25.19, *p* < 0.001). In both measurement points, seniors showed lower working memory than the two younger groups (T1: seniors-adults = −2.46 ± 0.63, *p* = 0.001; seniors-adolescents = −2.47 ± 0.60, *p* < 0.001; T2: seniors-adults = −3.46 ± 0.58, *p* < 0.001; seniors-adolescents = −3.40 ± 0.58, *p* < 0.001), while the two younger groups did not significantly differ from each other. On the other hand, *post hoc* comparisons showed that working memory decreased with measurement point in seniors (*t*_(26)_ = 2. 64, *p* = 0.014) but not adults or adolescents.

For depressive symptoms and level of perceived stress, a significant main effect of age group was found on CES-D (*F*_(2,75)_ = 9.26, *p* < 0.001, η_p_^2^ = 0.20) and PSS (*F*_(2,75)_ = 17.72, *p* < 0.001, η_p_^2^ = 0.32). Seniors scored significantly lower than the two younger groups (CES-D: seniors-adults = −6.98 ± 1.84, *p* = 0.002; seniors-adolescents = −4.45 ± 1.26, *p* = 0.003; PSS: seniors-adults = −10.14 ± 1.96, *p* < 0.001; seniors-adolescents = −8.63 ± 1.59, *p* < 0.001), while the two younger groups did not significantly differ from each other. No significant main effect of measurement point was found.

### Task performance

#### Participants’ identification accuracy

Participants’ identification accuracy was based on whether they could identify which tone quintets (i.e., XXXXX and XXXXY) were global deviants when they were asked to identify infrequent trials (see Table 4 for average rate). There was no age group × measurement point interaction. There was a significant main effect of age group (*F*_(2,75)_ = 14.71, *p* < 0.001, η_p_^2^ = 0.28). Seniors showed lower identification accuracy than the two younger groups (seniors-adults = −0.14 ± 0.03, *p* = 0.001; seniors-adolescents = −0.14 ± 0.03, *p* = 0.001), while the two younger groups did not significantly differ from each other. No significant main effect of measurement point was found.

**Table 4:** The mean (SD) of identification accuracy and counting errors

		T1	T2
Identification accuracy	Seniors	0.80 (0.24)	0.88 (0.18)
	Adults	0.97 (0.07)	0.98 (0.06)
	Adolescents	0.98 (0.06)	0.98 (0.05)
Counting errors	Seniors	8.65 (6.06)	6.69 (3.65)
	Adults	6.38 (6.96)	3.35 (2.82)
	Adolescents	5.44 (5.11)	3.79 (2.91)

#### Participants’ counting errors

Participants’ counting errors were quantified as the absolute difference between the actual and reported number of global deviants when they were asked to count the number of infrequent trials (see Table 4 for average discrepancy). There was no age group × measurement point interaction. There was a significant main effect of age group (*F*_(2,75)_ = 5.43, *p* = 0.006, η_p_^2^ = 0.13). Seniors showed larger counting errors than the two younger groups (seniors-adults = 2.80 ± 1.11, *p* = 0.041; seniors-adolescents = 3.05 ± 0.99, *p* = 0.009), while the two younger groups did not significantly differ from each other. There was a significant main effect of measurement point (*F*_(1,75)_ = 10.61, *p* = 0.002, η_p_^2^ = 0.12), where T1 showed larger counting errors than T2.

### ERPs

**Figure 3.**
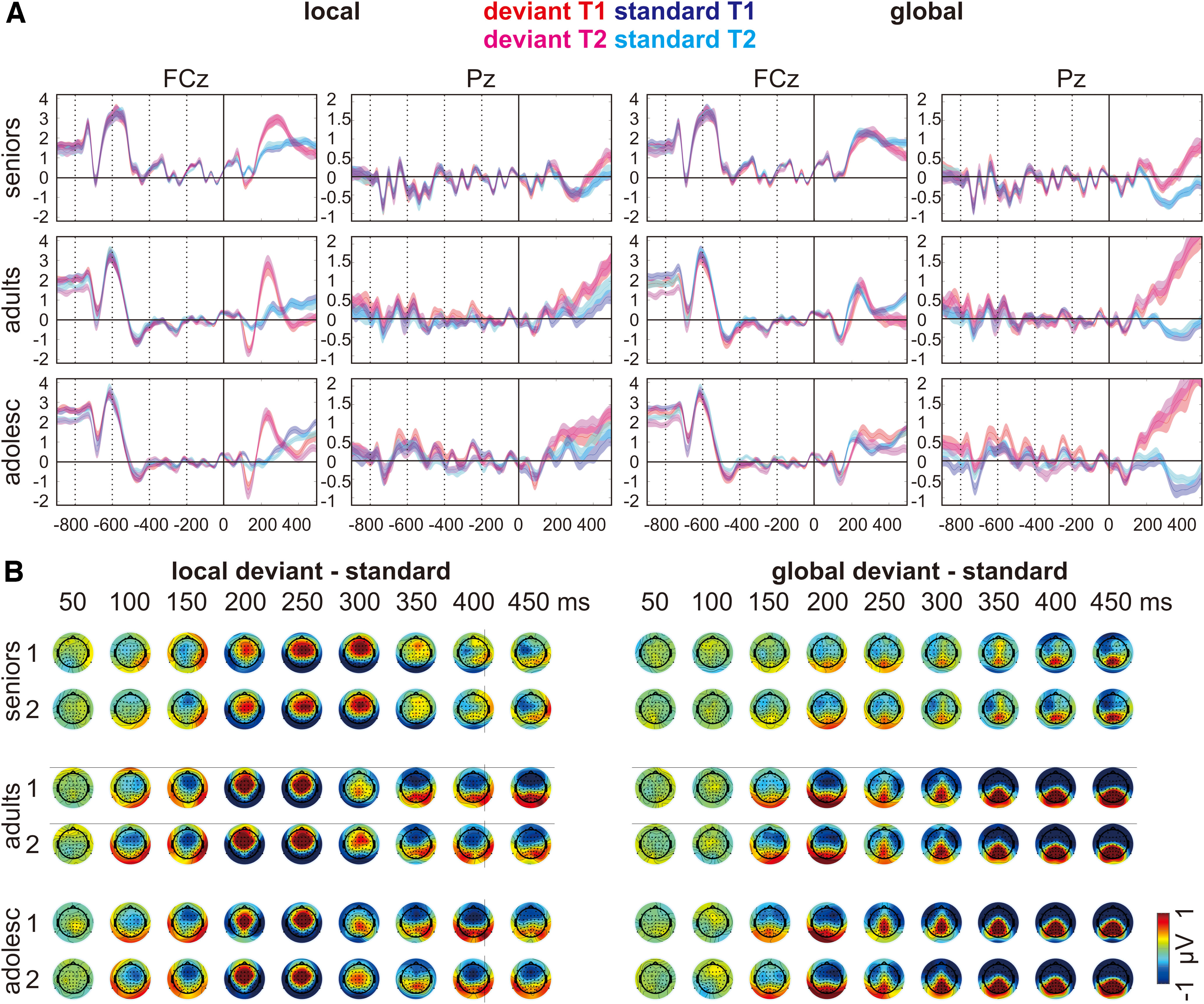
***A***, Grand averaged ERPs of local and global effects (i.e., deviant vs standard) on representative electrodes (i.e., FCz and Pz). Shaded area represents 1 SEM. Time 0 ms marks the onset of the probe. Dotted lines mark the onset of the four preceding tones. ***B***, Topographical distributions of local and global effects (i.e., deviant − standard) plotted from 50 to 450 ms after the onset of the probe.

**Figure 4.**
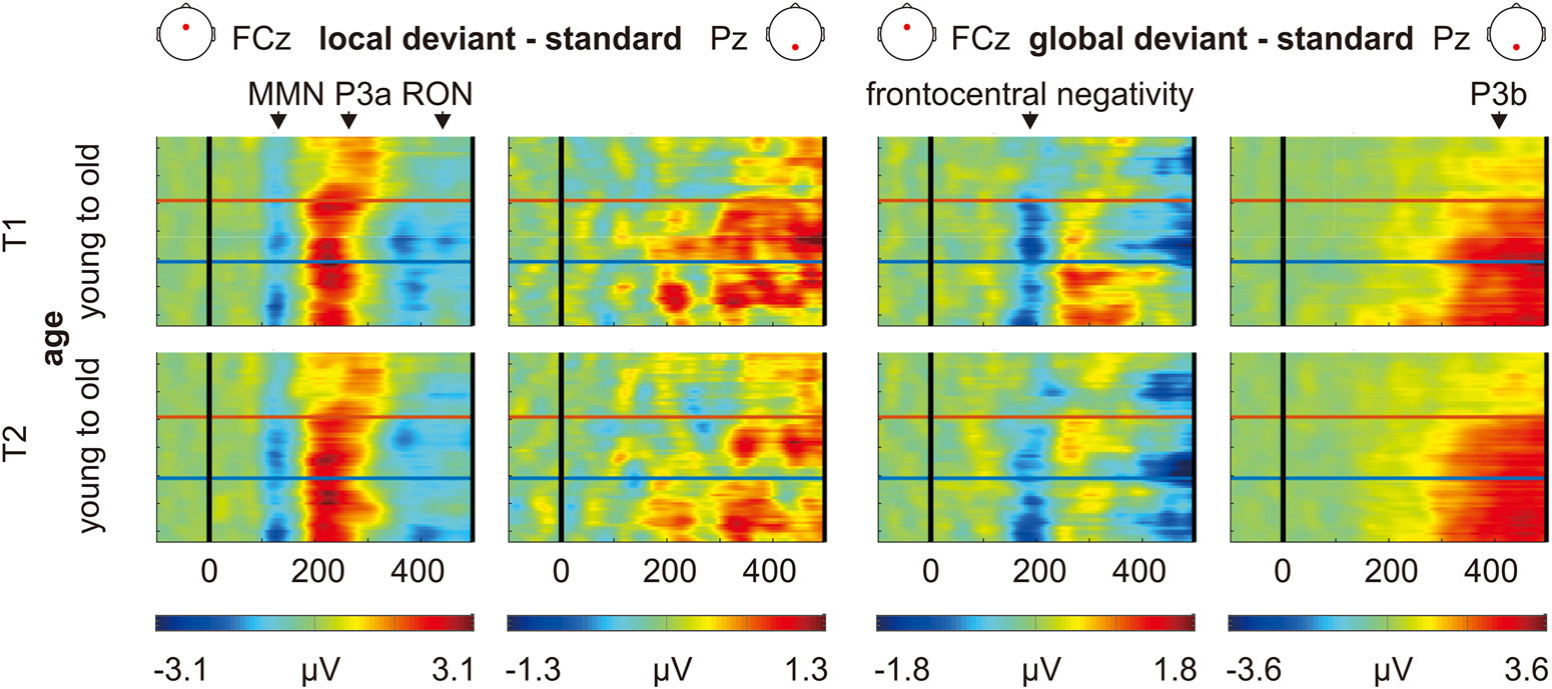
The 2-D (time-by-participant) views of the ERPs in which local and global effects (i.e., deviant − standard) are sorted along participants’ age, smoothed across neighboring participants using a rectangular (boxcar) moving average (smoothing width = 10), and color coded. The horizontal lines mark the boundaries of age groups (including seniors, adults, and adolescents).

Figure 3 shows the grand averaged ERPs and the topographical distributions of local and global effects in each age group and measurement point. Local effect included the MMN at around 100–150 ms, the P3a at around 200–300 ms, and the RON at around 350–450 ms. Global effect included the frontocentral negativity at around 150–200 ms and the P3b at around 250–450 ms. The 2-D (time-by-participant) views of the local and global ERPs sorted along participants’ age for each measurement point can be seen in Figure 4.

**Figure 5.**
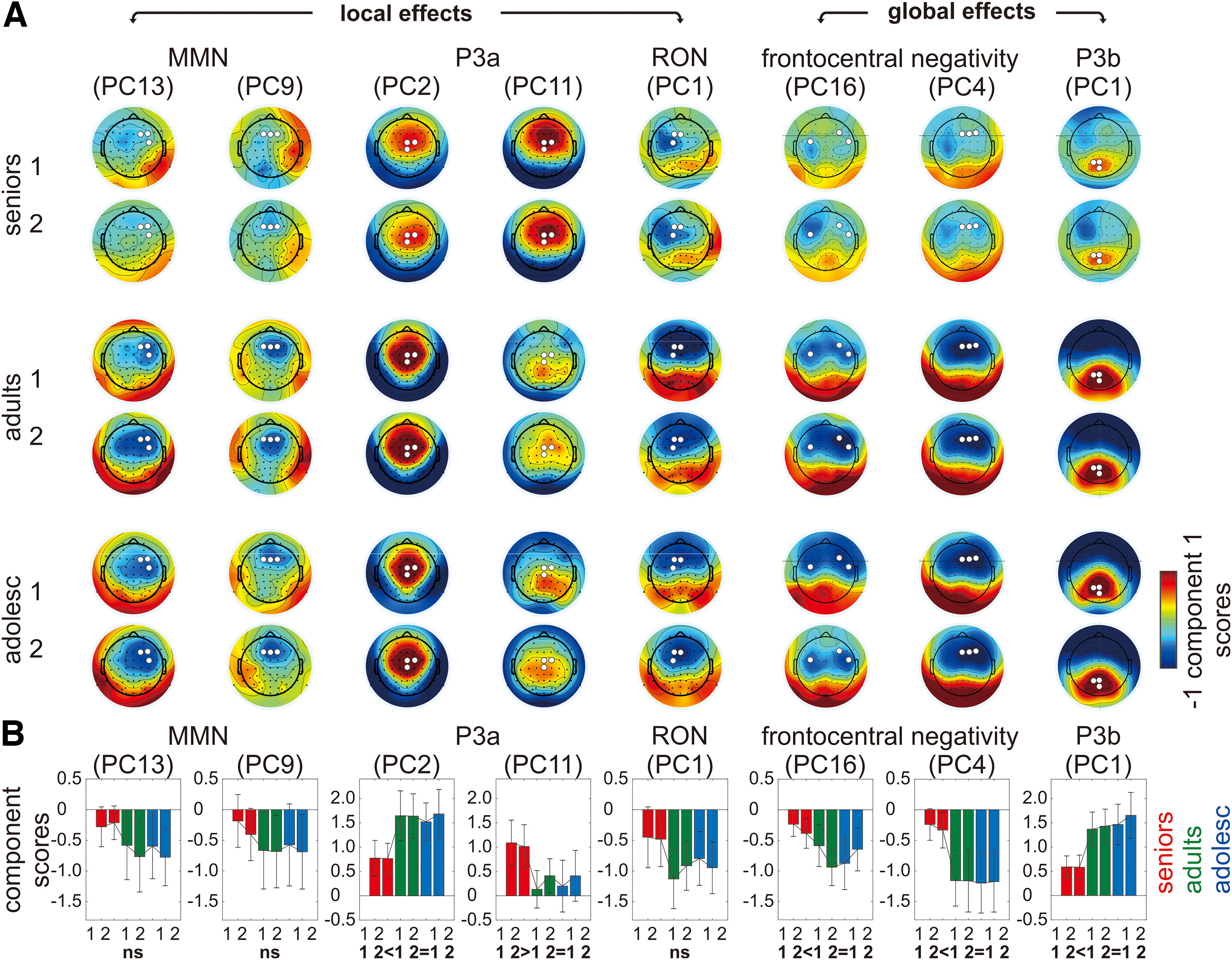
***A***, Topographical distributions of PCs for local effects (manifesting as the MMN, P3a, and RON) and global effects (manifesting as the frontocentral negativity and P3b) in each age group and measurement point. White dots mark three electrodes showing the most negative/positive responses across all conditions independent of experimental manipulation. ***B***, Component scores averaged across three maximum electrodes for each age group and measurement point. Error bar depicts one SD of the mean. Results of *post hoc* analysis are summarized in bold text at the bottom, where ns refers to non-significant difference.

Figure 5 shows the topographical distributions of PCs for local effects (manifesting as the MMN, P3a, and RON) and global effects (manifesting as the frontocentral negativity and the P3b) in each age group and measurement point. A 3 (age group: seniors, adults, adolescents) × 2 (measurement point: T1, T2) repeated measures ANOVA was performed on each component with Bonferroni correction, using an α level of 0.006 (i.e., 0.05/8).

#### Local effects

##### MMN

On either PC13 or PC9, there was neither interaction nor main effects, showing no evidence that the MMN changed across age groups and measurement points.

##### P3a

On PC2, there was no age group × measurement point interaction. There was a significant main effect of age group (*F*_(2,75)_ = 9.70, *p* < 0.001, η_p_^2^ = 0.21). Seniors showed smaller positivity than the two younger groups (seniors-adults = −0.87 ± 0.24, *p* = 0.002; seniors-adolescents = −0.83 ± 0.20, *p* < 0.001), while the two younger groups did not significantly differ from each other. There was no main effect of measurement point.

On PC11, there was no age group × measurement point interaction. There was a significant main effect of age group (*F*_(2,75)_ = 7.52, *p* = 0.001, η_p_^2^ = 0.17). Seniors showed larger positivity than the two younger groups (seniors-adults = 0.78 ± 0.21, *p* = 0.002; seniors-adolescents = 0.75 ± 0.24, *p* = 0.008), while the two younger groups did not significantly differ from each other. There was no main effect of measurement point.

Since both PC2 (peaking at 224 ms) and PC11 (peaking at 308 ms) represent the P3a, the result pattern of the repeated measures ANOVA indicated that there is an age-related shift in the latency of the P3a.

##### RON

On PC1, there was neither interaction nor main effects, showing no evidence that the RON changed across age groups and measurement points.

#### Global effects

##### Frontocentral negativity

On PC16, there was no age group × measurement point interaction. There was a significant main effect of age group (*F*_(2,75)_ = 5.59, *p* = 0.005, η_p_^2^ = 0.13). Seniors showed smaller negativity than the two younger groups (seniors-adults = 0.45 ± 0.15, *p* = 0.014; seniors-adolescents = 0.45 ± 0.14, *p* = 0.008), while the two younger groups did not significantly differ from each other. There was no main effect of measurement point.

On PC4, there was no age group × measurement point interaction. There was a significant main effect of age group (*F*_(2,75)_ = 13.71, *p* < 0.001, η_p_^2^ = 0.27). Seniors showed smaller negativity than the two younger groups (seniors-adults = 0.87 ± 0.19, *p* < 0.001; seniors-adolescents = 0.90 ± 0.17, *p* < 0.001), while the two younger groups did not significantly differ from each other. There was no main effect of measurement point.

Overall, the result pattern of the repeated measures ANOVA indicated that the frontocentral negativity was smaller in seniors than in the two younger groups.

##### P3b

On PC1, there was no age group × measurement point interaction. There was a significant main effect of age group (*F*_(2,75)_ = 15.97, *p* < 0.001, η_p_^2^ = 0.30). Seniors showed smaller positivity than the two younger groups (seniors-adults = −0.81 ± 0.17, *p* < 0.001; seniors-adolescents = −0.97 ± 0.18, *p* < 0.001), while the two younger groups did not significantly differ from each other. There was no main effect of measurement point.

To control the effect of task performance on the aforementioned results, we further performed two analyses of covariance (ANCOVA) using participants’ identification accuracy and counting errors (averaged across measurement points) as covariates. The result pattern remained unchanged except for the initial part of the frontocentral negativity (PC16) where the main effect of age group no longer reached significance.

**Figure 6.**
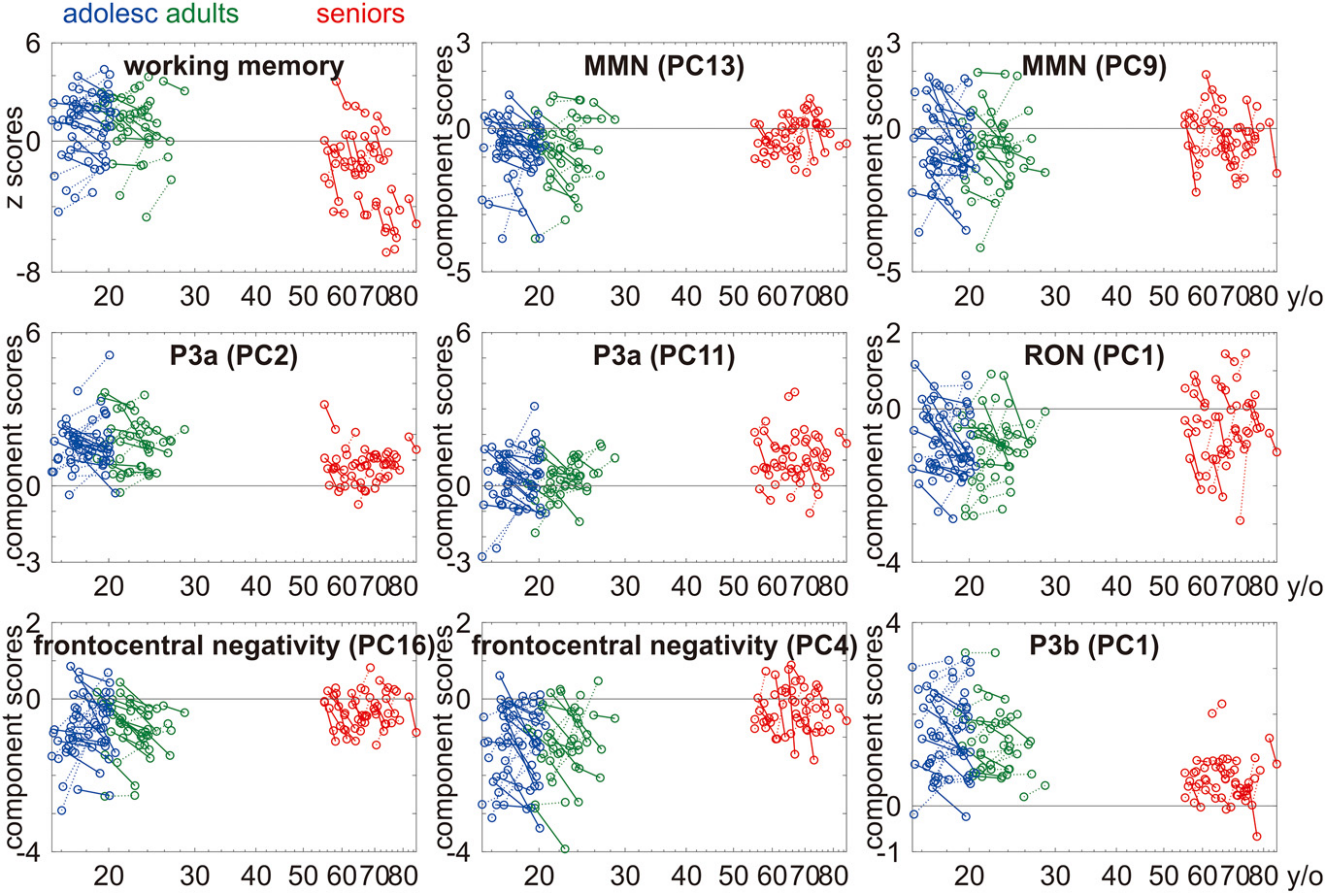
Age-related changes (*x*-axis in years of age on a base-10 logarithmic scale) in working memory (*y*-axis in *z* scores) and the ERPs (*y*-axis in component scores) at individual level. For ease of reference, increases with advancing age are plotted with dotted lines, whereas decreases with advancing age are plotted with solid lines.

Figure 6 summarizes the age-related changes in working memory and the local and global effects at individual level. From T1 to T2, seniors showed systematic decline in working memory but not in local and global effects.

### Correlation between the ERPs and working memory

**Figure 7.**
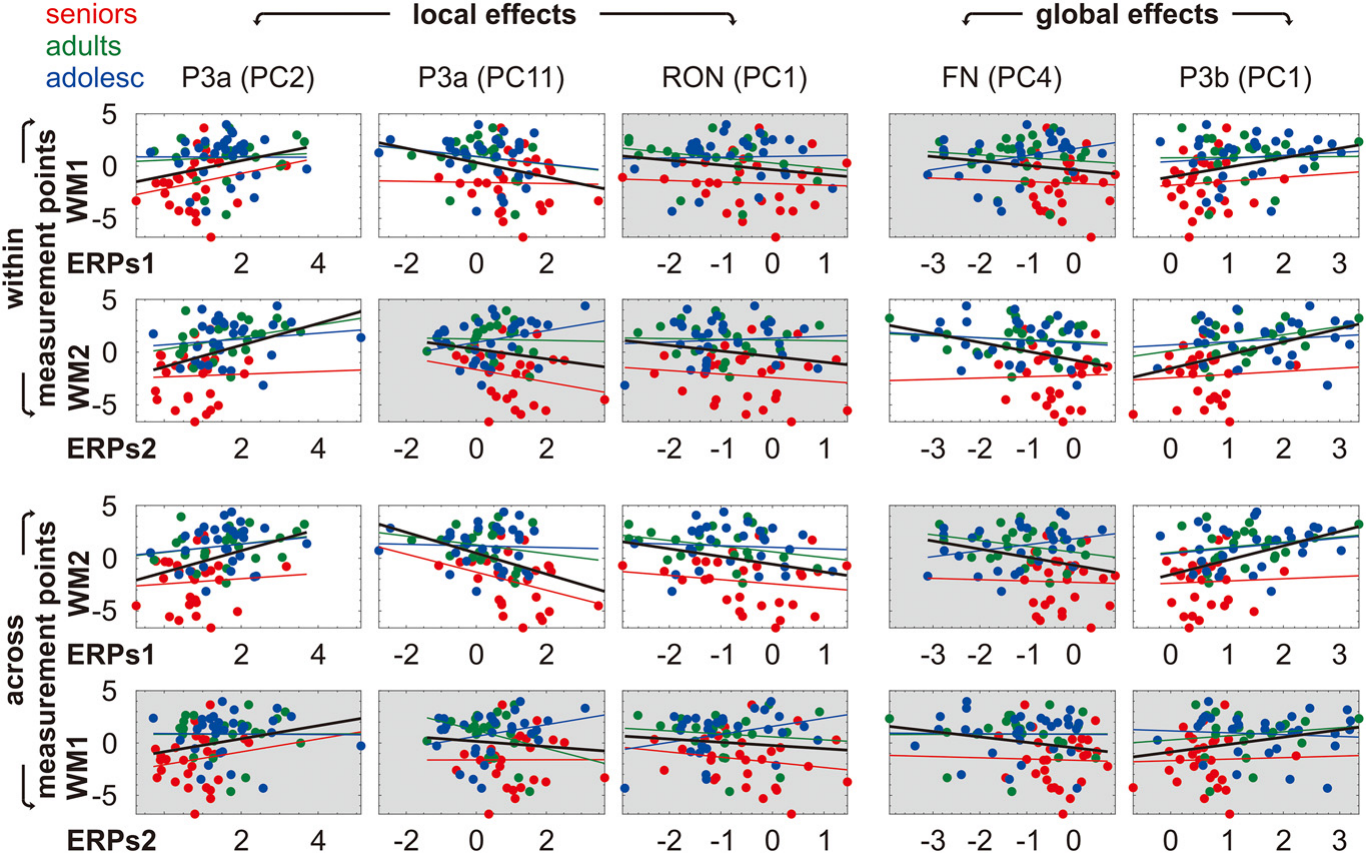
Scatterplots of the ERPs (in component scores) against working memory (in *z* scores). Least-squares lines by age group are plotted in different colors. Significant and non-significant correlations are respectively marked with white and gray background.

Partial correlations between the local and global effects versus working memory showed that, within both measurement points, the ERPs were correlated with working memory in similar manner (see Table 5 and Fig. 7, upper two rows). In T1, delayed P3a in local processing as well as smaller P3b in global processing were associated with worse working memory. In T2, delayed P3a in local processing as well as smaller frontocentral negativity and smaller P3b in global processing were associated with worse working memory.

**Table 5:** Partial correlations between the ERPs and working memory (*N* = 78) within and across measurement points controlling for depressive symptoms and level of perceived stress

			Local effects					Global effects		
			MMN	MMN	P3a	P3a	RON	FN	FN	P3b
			PC13	PC9	PC2	PC11	PC1	PC16	PC4	PC1
Within	ERPs1 WM1	*r*	0.17	−0.02	0.25 *	−0.25 *	−0.19	0.11	−0.08	0.27 *
		*p*	0.147	0.853	0.035	0.031	0.109	0.334	0.483	0.022
	ERPs2 WM2	*r*	−0.06	0.00	**0.34** **	−0.05	−0.14	−0.16	−0.27 *	**0.39** ***
		*p*	0.626	0.969	**0.003**	0.651	0.242	0.162	0.022	**0.001**
Across	ERPs1 WM2	*r*	0.12	−0.06	**0.31** **	**−0.35** **	**−0.30** **	−0.03	−0.17	**0.39** ***
		*p*	0.309	0.595	**0.006**	**0.002**	**0.009**	0.813	0.140	**0.001**
	ERPs2 WM1	*r*	0.09	0.01	0.20	−0.02	−0.07	−0.11	−0.17	0.21
		*p*	0.465	0.926	0.082	0.896	0.528	0.330	0.151	0.070

*p ≤ 0.05, **p ≤ 0.01, ***p ≤ 0.001; The boldface denotes *p* ≤ 0.05 after Benjamini–Hochberg procedure.

**Table 6:** Model summary of the hierarchical multiple regression for variables (including the ERPs, age group, and interaction) predicting working memory

			Stage one WM ∼ ERPs	Stage two WM ∼ ERPs + age group	Stage three WM ∼ ERPs + age group + interaction
Within measurement points	ERPs1 WM1	*R*^2^ change *F* change	0.18 *F*_(5,72)_ = 3.25 *p* = 0.011	0.10 *F*_(2,70)_ = 4.97 *p* = 0.010	0.07 *F*_(10,60)_ = 0.65 *p* = 0.768
	ERPs2 WM2	*R*^2^ change *F* change	0.25 *F*_(5,72)_ = 4.71 *p* = 0.001	0.18 *F*_(2,70)_ = 10.60 *p* < 0.001	0.05 *F*_(10,60)_ = 0.51 *p* = 0.879
Across measurement points	ERPs1 WM2	*R*^2^ change *F* change	0.32 *F*_(5,72)_ = 6.90 *p* < 0.001	0.15 *F*_(2,70)_ = 10.20 *p* < 0.001	0.06 *F*_(10,60)_ = 0.73 *p* = 0.698
	ERPs2 WM1	*R*^2^ change *F* change	0.10 *F*_(5,72)_ = 1.51 *p* = 0.198	0.14 *F*_(2,70)_ = 6.44 *p* = 0.003	0.08 *F*_(10,60)_ = 0.67 *p* = 0.746

Interestingly, we found a temporal asymmetry in the correlation across measurement points (see Table 5 and Fig. 7, lower two rows). On the one hand, T1 ERPs were correlated with T2 working memory, indicating that participants exhibiting delayed P3a and smaller RON in local processing as well as smaller P3b in global processing during T1 also showed worse working memory during T2. On the other hand, T2 ERPs were not correlated with T1 working memory. This asymmetric pattern seems robust since it survived the Benjamini–Hochberg correction.

The significant correlation between the ERPs and working memory could be driven by the wide range of working memory performance between age groups or one of the age groups (e.g., seniors as they showed working memory decline). To explore this issue, we performed a three-stage hierarchical multiple regression with working memory as the dependent variable. The ERPs showing significant correlation with working memory [i.e., P3a (PC2 and PC11), RON (PC1), frontocentral negativity (PC4), and P3b (PC1)] were entered at stage one, age group was entered at stage two, and the ERPs × age group interaction was entered at stage three of the regression model (Table 6). At stage one, the ERPs contributed significantly to the regression model within measurement points and showed a temporal symmetry across measurement points. Introducing age group to the regression model at stage two explained an additional 10–18% of the variance in working memory and all of the changes in *R*^2^ were significant. Introducing the ERPs × age group interaction to the regression model at stage three explained an additional 5–8% of the variance in working memory but none of the changes in *R*^2^ were significant. On the other hand, the interpretation of the standardized β coefficients should be limited given the small sample size. The result pattern indicated that the correlation between the ERPs and working memory was driven by the wide range of working memory performance between age groups, not one of the age groups.

## Discussion

Here, we used the auditory local-global paradigm to examine how the hierarchical predictive processing might change alongside working memory in normal aging. The three-year follow-up showed that seniors consistently diverged from the two younger groups in cognitive functions and the ERPs. Concerning working memory, seniors scored lower than the two younger groups in both measurement points. Moreover, within three years’ time, working memory declined in seniors but not the two younger groups. Concerning electrophysiological responses in the auditory local-global paradigm, seniors exhibited largely preserved local processing (consisting of comparable MMN, delayed P3a, and comparable RON) but significantly compromised global processing (consisting of suppressed frontocentral negativity and suppressed P3b). Unlike working memory, electrophysiological responses did not change with the passing of time, indicating that it is a stable pattern of neural activation within individuals. Correlation analysis further showed that the stable pattern of neural activation signaling prediction errors is related to working memory measured concomitantly. Moreover, there was a correlation between earlier predictive processing and later working memory but not between earlier working memory and later predictive processing. The results suggested that the hierarchy-selective attenuation of prediction errors is likely a precursor of deteriorated working memory.

### Working memory deteriorates with advancing age

Our cross-sectional analysis supported the common report of an age-related decline in working memory (Salthouse, 1991; Fisk and Warr, 1996; for review, see Salthouse, 1990, 1994). Our longitudinal analysis further showed that, within three years’ time, we can already observe working memory deterioration in seniors but not the two younger groups. This is in line with previous research of longitudinal changes in older individuals showing significant decline in verbal working memory over three to four years’ time (Hultsch et al., 1992; Rieckmann et al., 2017). Nevertheless, we measured participants’ working memory with three subtests, including Digit Span, Letter-Number Sequencing, and Arithmetic in WAIS-IV. Therefore, the observed loss of working memory in older age might not necessarily reflect a monolithic decay. For example, although the three subtests all measure the construct of working memory, there seems to be considerable heterogeneity in when different scores rise and fall with age (Hartshorne and Germine, 2015). Exploratory analyses on the three subtests also revealed that the age group × measurement point interaction was significant on Digit Span (*F*_(2,75)_ = 6.95, *p* = 0.002, η_p_^2^ = 0.16) but marginal on Letter-Number Sequencing (*F*_(2,75)_ = 2.40, *p* = 0.098, η_p_^2^ = 0.06) and Arithmetic (*F*_(2,75)_ = 2.60, *p* = 0.081, η_p_^2^ = 0.07). Therefore, the overall deterioration in older age could result from different amounts of age-related changes in different subcomponents of working memory.

### Age-related attenuation of prediction errors is a stable pattern

The auditory local-global paradigm allowed the observation of a series of local effects representing sensory (i.e., lower-level) prediction errors (including the MMN, P3a, and RON) as well as global effects representing contextual (i.e., higher-level) prediction errors (including the frontocentral negativity and P3b). On the one hand, the MMN/P3a/RON complex provides a neurophysiological index of the cascade of three main processes involved in involuntary attention control to violations of local regularity, including automatic change detection, orienting of attention, and recovery from distraction or reorienting of attention (Schröger and Wolff, 1998; Schröger et al., 2000; Berti and Schröger, 2003; Berti et al., 2004; Berti, 2008; Horváth et al., 2008). On the other hand, the presence of the frontocentral negativity and P3b requires subjective awareness of the global regularity of the stimuli, which could disappear when participants were mind-wandering, doing a visual interference task, or in a vegetative state (Bekinschtein et al., 2009). Hsu et al. (2021) previously found that, in normal aging, while the detection of local deviancy seems largely preserved (as seniors showed comparable MMN; see also Avancini et al., 2022), the detection of global deviancy is clearly compromised (as seniors showed suppressed P3b). The three-year follow-up confirmed the aforementioned findings of the hierarchy-selective attenuation of prediction errors in older brains, suggesting that seniors can perform automatic integration of the auditory surroundings but cannot transfer it into conscious perception as efficiently as younger ones.

The lack of interaction with measurement points further highlights the stability of the ERPs. None of the age groups exhibited significant changes in the ERP responses with the passing of time. Visual inspection of the ERP waveforms in the three age groups also revealed them to be largely overlapping between measurement points. This accords with previous research reporting relatively high test-retest reliability, for example, of the P3 to auditory oddballs across a three-year interval in old adults (Sandman and Patterson, 2000), across a one-week interval in young adults (Debener et al., 2002), and across a one-year interval in old and young adults (Walhovd and Fjell, 2002). Our results extend the P3 findings and suggest that a range of ERPs signaling prediction errors remain stable across a time span of three years. While the reliability of the local and global neural responses indicates their potential utility as a clinical marker, it also points to the need to adopt even longer follow-up period to model age-related trajectories of predictive processing.

### Neural responses to prediction errors were correlated with working memory

The current research demonstrated a robust correlation between the ERPs signaling prediction errors and working memory within measurement points. Participants exhibiting delayed P3a in local processing and suppressed P3b in global processing showed worse working memory measured concomitantly. The concomitant correlation can be expected from a neuropsychological model for the P3 (Polich, 2007) suggesting that the P3a arises from a stimulus-driven frontal mechanism engaged to evaluate incoming stimuli in working memory (Berti, 2016) whereas the P3b reflects the temporoparietal processing of these stimuli related to the updating of working memory. Also, previous research commonly documented an age-related delay and reduction in P3a and P3b (Porcaro et al., 2019; ElShafei et al., 2022) and reported a concomitant link between P3a amplitude versus amnestic subtypes of mild cognitive impairment (Correa-Jaraba et al., 2018) as well as P3b amplitude versus performance in a logical memory task (Porcaro et al., 2019).

Interestingly, we also found a temporal asymmetry in the correlation across measurement points. Specifically, there was a correlation between earlier ERPs and later working memory but not between earlier working memory and later ERPs. The temporal asymmetry indicates that the attenuation of prediction errors is likely a precursor of poor working memory. It is as if the brains of seniors and the two younger groups are already in different categories of stable states, which happens before further deterioration of working memory takes place. A particularly interesting question for future research is whether the hierarchy-selective attenuation of prediction errors might be used to predict working memory decline in the long run before any clinically relevant symptoms would manifest in the aging population.

### Limitations and future directions

The current research is subject to the following limitations. First, we did not acquire audiometric data from the participants. While all participants reported no diagnosis of hearing impairments, future research should include objective measurement to determine whether potential differences in hearing thresholds might contribute to our findings. Second, the sample size per group was <30 because of dropouts from T1 to T2. While we found the age-related attenuation of prediction errors which remained stable across measurement points, it is important for future research to investigate whether the age-related attenuation of prediction errors might interact with measurement points when the sample size per group increases. Lastly, although the current research showed that the hierarchy-selective attenuation of prediction errors is related to deterioration in working memory, highlighting the potential of signal attenuation as a biomarker for cognitive decline, still little is known about its onset and course in the brain. When does it start and how does it develop? The inclusion of a middle-aged sample in future research is expected to reveal the shape of the developmental trajectory.

## Synthesis

Reviewing Editor: Alexander Soutschek, Ludwig-Maximilians-Universitat Munchen Decisions are customarily a result of the Reviewing Editor and the peer reviewers coming together and discussing their recommendations until a consensus is reached. When revisions are invited, a fact-based synthesis statement explaining their decision and outlining what is needed to prepare a revision will be listed below. The following reviewer(s) agreed to reveal their identity: NONE. Note: If this manuscript was transferred from JNeurosci and a decision was made to accept the manuscript without peer review, a brief statement to this effect will instead be what is listed below.

The authors analyze data from a global-local oddball task and compare three age groups (adolescents, adults, and seniors). They also assess working memory performance using three different scales. Behaviorally, seniors showed reduced task performance. For local violations of environmental statistics, they found no significant differences across groups in PCA components loading on MMN and RON, but they did find significantly reduced early P3a (224ms) and significantly increased late P3a (308ms) suggesting delayed P3a in seniors. Following global violations of expectations they found reduced frontocentral negativity and P3b in seniors. Changes in P3a and P3b related to worse working memory and early reductions in P3a and RON correlated with later WM impairments, but ERPs recorded at follow up were not correlated with earlier WM impairments suggesting that ERP change may be a precursor of WM decline.

Overall, the paper is clearly written and the work is very extensive as it investigates local and global violations and 5 components (MMN, P3a, RON, P3b and frontocentral negativity) in a longitudinal design in three groups. I found the results interesting and appreciated the hard work the authors did. However, I have some concerns that need addressing. I hope you will find my comments helpful and constructive:

Major points:

1.Introduction, p.6: You highlight previous findings from Hsu et al (2021) showing intact MMN and impaired P3b and argue that this reflects impaired low-level PEs. However, you later consider P3b to be a global signal (i.e., reflecting high-level PEs). I tend to agree with this later view also in light of other recent findings suggestion that P3 relates to PEs about environmental volatility (i.e., a higher level; e.g., https://www.nature.com/articles/s41537-022-00302-3a and https://www.medrxiv.org/content/10.1101/2022.12.20.22283712v1). I do not see how this fits the narrative that low-level PEs are impaired. Could you explain that? Along those lines it might be worthwhile to discuss these model-based results in relation to you findings.

2. Sample: Were individual hearing thresholds determined? Or is there any insurance that elder participants heard the full range of tones? since perceived tone frequencies and loudness are impacted by aging. If not this is an important limitation to mention.

3. Preprocessing, p.12: Why did the PCA procedure change from T1 to T2? This should be kept identical. Was the PCA done per group or on all subjects?

4. Preprocessing: Also regarding the PCA, I do not understand why you investigated PCs rather than looking at the ERPs directly, since you ultimately included a large number of components there does not seem to be much of an advantage in terms of reducing the number of tests, but you do sacrifice interpretability as each PCA component is a mixture of ERP components.

5. Table 4: It looks like there were practice effects (e.g., higher accuracy for seniors in t2t2), can you describe training procedures for participants? Is this a problem?

6. Statistical analysis: I noticed that the variance in the old age group is much larger than in the other two groups, it is unclear whether you tested/accounted for unequal variances.

3. Statistical analysis: Since there were performance differences between the old age group and the younger groups, it is unclear whether ERP changes (e.g., in P3b) result from seniors paying less attention to the task. I would recommend to repeat the ERP analysis and taking performance differences as a covariate into account and test which results cannot be explained by performance differences.

7. Results, Figure 5, Can you report and/or show the correlation between PCs and WM within each group.

8. Limitations: You do not discuss limitations of your work in the limitation section, such as the duration of the follow-up which may have precluded you from identifying changes in ERPs and a group-by-time interaction, relatively small sample size per group, and possibly that results could be confounded by performances differences and hearing impairments (at least given the current description of your analyses).

Minor points:

1. Why was a jittered ITI chosen? Does this not lead to some tones being both a deviant with respect to frequency AND onset?

2. Figure 2: the median lines and mean asterisks should be offset to increase readability of this figure. Please also highlight significant differences in the figure.

3. P. 22, RON section: “On PC1, there was neither interaction nor main effects, suggesting comparable RON across age groups and measurement points” This is a bit nitpicky, but the absence of a significant effect should not be interpreted as evidence for the absence.

4. Please report p-values in tables.

5. Did you use the total score of the digit span scale or just the backward score, since this score is stronger related to WM, whereas forward rather related to short term memory?
